# Effect of Atmospheric Room Temperature Plasma on the Volatile Profile of Laurel: Optimization by HS-SPME/GC-MS Analysis with Application in a Ready-to-Use Broth Model

**DOI:** 10.3390/foods15132346

**Published:** 2026-07-02

**Authors:** Martha Mantiniotou, Vassilis Athanasiadis, Dimitrios Kalompatsios, Eleni Bozinou, George Ntourtoglou, Vassilis G. Dourtoglou, Stavros I. Lalas

**Affiliations:** 1 Department of Food Science and Nutrition, University of Thessaly, Terma N. Temponera Street, 43100 Karditsa, Greece; mmantiniotou@uth.gr (M.M.); vaathanasiadis@uth.gr (V.A.); dkalompatsios@uth.gr (D.K.); empozinou@uth.gr (E.B.); 2 Department of Wine, Vine and Beverage Sciences, University of West Attica, Agiou Spyridonos Street, Egaleo, 12210 Athens, Greece; gntourtoglou@uniwa.gr (G.N.); vdourt@uniwa.gr (V.G.D.)

**Keywords:** laurel powder, cold plasma, response surface methodology, HS-SPME, GC-MS, volatile compounds, eucalyptol, α-pinene, broth model, food application

## Abstract

Laurel (*Laurus nobilis* L.) is a characteristic species of the Mediterranean flora, valued for its medicinal, aromatic, and culinary uses. Many of these properties are attributed to its volatile constituents. In this study, the effect of Atmospheric Room Temperature Plasma (ARTP) pretreatment on laurel powder was evaluated, with emphasis on volatile recovery and food application. Volatile extraction was optimized using headspace solid-phase microextraction (HS-SPME) coupled with gas chromatography–mass spectrometry (GC-MS), investigating key parameters such as salt concentration, extraction temperature, equilibration time, extraction time, and fiber type. Subsequently, critical ARTP variables (nitrogen flow, treatment duration, treatment distance, substrate thickness, and plasma power) were optimized. Response Surface Methodology was applied in both optimization processes. The results demonstrated that fiber type was the most influential factor for volatile recovery, with extraction temperature also exerting a significant effect. A more nuanced pattern emerged during ARTP pretreatment, where moderate plasma intensities enhanced the recovery of several key volatiles. This trend was not uniform across compounds, indicating that plasma-induced microstructural changes interact with the physicochemical properties of individual analytes. To demonstrate food relevance, a ready-to-use broth model prepared with laurel powder confirmed improved headspace transfer of characteristic volatiles following ARTP treatment. Taken together, these findings suggest that ARTP can serve as a practical, non-thermal pretreatment for improving volatile release, supporting its potential use in the food, pharmaceutical, and cosmetic industries.

## 1. Introduction

*Laurus nobilis* L. is a flowering, evergreen tree or large bush, often used as an aromatic, spicy, and medicinal plant. It is native to the southern Mediterranean region of Europe [1]. It is also known as sweet bay, bay laurel, Roman laurel, or daphne, belonging to the Lauraceae family [2]. Laurel leaves are commonly used in cooking to impart fragrance and flavor to meat, fish, broths, and vegetables [3,4], and they also exhibit cleansing properties [5]. Previous studies indicate that laurel leaves may contain up to 270 volatile compounds, with the predominant ones being 1,8-cineole or eugenol (22–56%), linalool (0.9–26.9%), α-terpinyl acetate (4.5–18.2%), α-pinene (2.2–15.9%), β-pinene (1.9–15.3%), sabinene (4.5–12.7%), α-terpineol (0.9–12.0%), and terpineol-4 (0.9–4.1%) while limonene is also present [6,7,8]. Essential oils are often recognized as natural preservatives that inhibit food rotting via their antibacterial properties [9]. In Mediterranean regions, laurel has long been used in ethnomedicine to address a lack of appetite, abdominal discomfort, and fever. The essential oil and different preparations of laurel leaves and bark may alleviate headaches, migraines, hyperglycemia, bacterial and fungal infections, and stomach ulcers. They possess anti-inflammatory and antioxidant effects [6]. The essential oil of the leaves is used in the treatment of rheumatism and skin rashes, as a wound healing agent, and in the cosmetics sector [6,10].

The analytical characterization of laurel volatiles relies predominantly on gas chromatography–mass spectrometry (GC-MS), typically preceded by headspace solid-phase microextraction (HS-SPME). HS-SPME is widely adopted due to its solvent-free nature and high sensitivity, yet its performance is strongly dependent on operational parameters such as fiber coating, extraction temperature, salt concentration, equilibration time, and extraction duration [11]. Because these factors often interact in non-linear ways, empirical optimization becomes challenging and may lead to biased or irreproducible results. Response Surface Methodology (RSM) provides a structured and statistically robust framework for identifying optimal extraction conditions and understanding factor interactions.

In parallel, Atmospheric Room Temperature Plasma (ARTP) has emerged as a promising non-thermal pretreatment for enhancing the extraction of bioactive compounds from plant matrices. ARTP and related cold plasma technologies generate reactive nitrogen species (RNS) under ambient conditions, enabling micro-etching of plant surfaces, disruption of cell wall structures, increased permeability, and improved release of intracellular constituents [12,13,14]. Recent studies have demonstrated that plasma pretreatment can significantly enhance the extraction of phenolics, antioxidants, and other phytochemicals from botanical materials, including *Moringa oleifera* leaves, coffee grounds, peach peels, and edamame [15,16,17,18]. Importantly, the use of nitrogen as a process gas minimizes oxidative degradation, making ARTP particularly suitable for heat-sensitive phytochemicals, such as volatile terpenoids.

Despite these advantages, a critical knowledge gap exists in the literature: no previous study has evaluated the effect of ARTP pretreatment on the volatile profile of laurel powder, nor has any work combined HS-SPME optimization with plasma-assisted enhancement of volatile recovery. Equally important, the influence of ARTP on volatile compound transfer in real food systems remains unexplored, leaving its practical relevance for culinary or industrial applications uncertain.

Therefore, the present study aimed to (i) optimize HS-SPME/GC-MS conditions for the extraction of key laurel volatiles using RSM, (ii) investigate the effect of ARTP pretreatment on volatile release by modeling five plasma parameters (distance, thickness, power, flow, and duration), and (iii) evaluate the practical impact of ARTP-treated laurel powder in a ready-to-use broth model to assess volatile compounds transfer under realistic food preparation conditions. This integrated approach provides new insight into plasma-assisted enhancement of aromatic plant materials, supporting the development of sustainable pretreatment strategies for natural flavoring agents.

## 2. Materials and Methods

### 2.1. Laurel Leaves Material Handling

Laurel leaves were collected from a local farmer in the Kavala region of Eastern Macedonia, Greece, coordinates 40°59′18.0″ N, 24°36′57.0″ E according to Google Earth (ver. 10.91.0.1 from Google Inc. (Cambridge, MA, USA). The leaves were allowed to dry naturally at ambient temperature for 10 d. The moisture content was determined in triplicate using a Moisture Analyser DAB 200-2, provided by Kern (Frankfurt, Germany), initially on day 1 and finally on day 10. Moisture content was 51.7 ± 2.3% (*n* = 3). The leaves were air-dried under ambient conditions, which reduces moisture but does not fully dehydrate the material. Then they were ground into fine powder using a laboratory electric mill (SilverCrest Coffee Grinder SKME150; Kompernass Handels GmbH, Bochum, Germany). The powder then underwent a sieving process with an Analysette 3 PRO apparatus from Fritsch GmbH (Oberstein, Germany), with an average particle diameter of 459 μm.

### 2.2. Chemicals and Reagents

Sodium chloride (NaCl ≥ 99.0% *w*/*w*) was supplied by Penta (Prague, Czech Republic), and 2-octanol by Sigma-Aldrich (Darmstadt, Germany). *n*-Alkane standard (C7–C30) used for the calculation of retention indices (RI) was a certified reference material (TraceCERT^®^, Supelco, Merck KGaA, Darmstadt, Germany; Product Code: 49451‑U). Ethanol (absolute, 99.8% *v*/*v*) was purchased from Fisher Chemical (Pittsburgh, PA, USA). Deionized water used in all experiments was prepared with a mixed-bed ion-exchange resin column, ensuring conductivity below 1 μS/cm under standard flow rate and operating pressure conditions.

### 2.3. Experimental Design

A custom design of experiments (DOE) was constructed using JMP^®^ Pro 16 (SAS Institute Inc., Cary, NC, USA) to optimize the headspace solid-phase microextraction (HS-SPME) conditions for the volatile analysis of laurel leaf samples. Five factors were included: one categorical factor (fiber type, five levels: PDMS, PDMS/DVB, DVB/CAR/PDMS, CAR/PDMS, and PA) and four continuous factors (extraction temperature, NaCl concentration, equilibration time, and extraction time). The continuous factors were investigated at three coded levels (−1, 0, +1), corresponding to 20, 40, and 60 °C for temperature; 0, 15, and 30% *w*/*v* for NaCl; 10, 20, and 30 min for equilibration; and 10, 20, and 30 min for extraction. These factors were chosen as they are of paramount importance for the HS-SPME procedure. A total of 28 experimental runs with two center points were conducted, with the experimental factors and their corresponding levels summarized in Table 1; detailed information on the experimental runs is provided in Table S4.

The statistical model incorporated main effects for all factors, quadratic terms for the continuous variables, and one targeted two-way interaction (temperature × NaCl). The design consisted of 20 experimental runs, arranged in five blocks (corresponding to experimental days), with four runs per block. Center points (all continuous factors at level 0) were distributed across different fibers to provide an estimate of pure error and to assess curvature. To reduce errors, the sequence of the runs inside each block was randomized.

The primary response quantified was the overall GC-MS peak area of volatile compounds, whereas secondary responses included specific marker volatiles (e.g., 1,8-cineole, linalool, eugenol) and the recovery of the internal standard (2-octanol). The internal standard (2-octanol) was evaluated across all runs and showed low variability (RSD < 8%), confirming analytical stability. It was therefore used only for relative normalization and not as an absolute quantitative reference. Retention indices (RI) were also calculated for all detected volatiles using a C7–C30 *n*-alkane standard (Supelco 49451-U) to support compound identification. The DOE framework made it easier to find the most important elements and interactions that affect volatile extraction, while maintaining the overall number of GC-MS runs within a reasonable range. The full experimental design matrix, including all factor combinations and response values, is provided in Supplementary Table S1. The complete ANOVA statistics and regression equations for the HS-SPME optimization models are presented in Supplementary Tables S2 and S3, respectively, offering full transparency regarding model structure and significance.

### 2.4. Sample Preparation and Gas Chromatography–Mass Spectrometry (GC-MS) Analysis

Laurel leaf samples were ground to a fine powder, and 1.0 g portions were weighed into 25 mL glass vials. Each vial was supplemented with 10 mL of deionized water and sodium chloride according to the experimental design (0–30% *w*/*v*). The mixture was spiked with 2-octanol (final concentration 2 mg/L) as an internal standard. Vials were sealed with PTFE/silicone septa and placed in a thermostated oil bath at the designated extraction temperature (20–60 °C). For the oil baths, stirring hotplates with integrated thermometers (Agro-LAB M3 D) were used. After an equilibration period of 10–30 min, an SPME fiber (Supelco, Bellefonte, PA, USA), preconditioned at 270 °C for 30 min (according to the manufacturer’s recommendations), was exposed to the headspace for 10–30 min under continuous stirring at 250 rpm.

Following extraction, the fiber was manually inserted into the injector of an Agilent 7890A gas chromatograph coupled to a 5975C mass selective detector (Agilent Technologies, Folsom, CA, USA). Chromatographic separation was achieved on a DB-1 capillary column (30 m × 320 μm × 0.25 μm) with helium as carrier gas at 1.5 mL/min. The injector was operated in splitless mode at 240 °C. The oven program was: 40 °C for 5 min, ramped at 2 °C/min to 140 °C, then at 10 °C/min to 240 °C, held for 10 min. The total run time was 75 min. The MS was operated in electron impact mode (69.9 eV), scanning *m*/*z* 29–350, with source and quadrupole temperatures of 230 °C and 150 °C, respectively. Operating the MS source at 230 °C served three purposes: it minimized sample condensation, prevented degradation of fragile compounds, and created a stable environment for efficient ionization. Meanwhile, a quadrupole temperature of 150 °C was selected to maintain the thermal stability of the electronics responsible for the radio-frequency voltage, a key factor in preserving mass accuracy and signal consistency.

Compound identification was performed by comparing the acquired mass spectra with the NIST11 and Wiley W8N08 libraries. A minimum spectral match similarity index of ≥80% was used to confirm compound identity. Quantification was conducted in a semi-quantitative manner, based on normalization of individual peak areas to the internal standard (2-octanol, 2 mg/L). No calibration curves were employed; therefore, results are expressed as milligrams of 2-octanol equivalents per liter of sample solution (mg 2-octanol eq/L). This approach enables relative comparison of volatile recovery across treatments, while maintaining analytical consistency and minimizing matrix effects.

Retention indices (RIs) were calculated according to the linear Kovats method for temperature-programmed gas chromatography. A homologous series of *n*-alkanes (C7–C30) was injected under identical chromatographic conditions, and the RI of each analyte was obtained by linear interpolation between the retention times of the two adjacent alkanes (C_z_, C_z+1_) that bracketed the analyte. The combined use of mass spectral matching and RI agreement with the literature values ensured high-confidence identification of all volatile constituents.

### 2.5. Atmospheric Room Temperature Plasma Process

An ARTP apparatus was utilized to conduct the ARTP process, produced via Piezoelectric Direct Discharge (PDD^®^) technology using the Piezobrush^®^ PZ3-i device from Relyon Plasma GmbH (Regensburg, Germany). The pretreatment procedure employed ARTP and PDD^®^ processes, which produce plasma by emitting high electric fields from a piezo-ceramic transformer, converting a low input voltage (24 V DC) into a high-voltage discharge within the process gas. Nitrogen served as an operating medium to activate reactive nitrogen species, which included charged particles, radicals, and ultraviolet photons.

For each experimental run, 1.0 g of laurel powder was evenly distributed in a sterile 90-mm Petri dish. The sample remained stationary during treatment to ensure consistent exposure geometry. The plasma nozzle was positioned at the designated distance above the powder surface, and nitrogen flow was maintained according to the experimental design. Surface temperature was monitored immediately after each treatment using a non-contact infrared thermometer and remained below 40 °C in all cases, confirming the non-thermal nature of the process. No visible charring, discoloration, or agglomeration of the powder was observed following ARTP exposure.

A Central Composite Design (CCD) was constructed to optimize five plasma pretreatment parameters. The design included 28 runs, covering factorial, axial, and central points. Statistical analysis revealed high power (>0.95) for all primary effects and interactions, while moderate power (~0.27) was observed for quadratic terms. The assessment of effectiveness and diagnosis of forecast variance confirmed the robustness of the model, indicating D-effectiveness of 41.4 and G-effectiveness of 72.2. This design established a robust framework for response surface modeling and the optimization of plasma-induced effects on the recovery of volatile compounds. The pretreatment effect on the recovery of volatile compounds was relied on the optimization of five independent factors: The distance from the material surface to the plasma source (*D*, mm) is *X*_1_, the material thickness (*T*, mm) is *X*_2_, the plasma power (*P*, %) is *X*_3_, the nitrogen flow rate (*F*, L/min) is *X*_4_, and the treatment duration (*t*, min) is *X*_5_. Each variable was evaluated at three levels: low (−1), medium (0), and high (+1), as seen in Table 2. The thickness of each sample was calculated utilizing a thickness gauge. A total of 28 experimental trials, including two central points, were conducted in duplicate. The average values of each answer were recorded for model fitting and analysis. The complete CCD with coded and actual factor levels, along with the measured responses for all 28 runs, is presented in Supplementary Table S4. The full ANOVA statistics for the ARTP RSM models are provided in Supplementary Table S5, while the corresponding regression equations in coded units are reported in Supplementary Table S6.

### 2.6. Broth Preparation

A simple ready-to-use broth (0.5% *w*/*v* NaCl, 0.2% *w*/*v* citric acid; pH ~5.5) was prepared with laurel powder at 0.5 g per 100 mL. Broths were heated at 90 °C for 15 min and held at 60 °C for 30 min to simulate culinary infusion. Post heating, broths were centrifuged to separate the supernatant. Control and ARTP-treated powders were processed identically. Following clarification, 10 mL aliquots were analyzed by HS-SPME/GC-MS as described (2-octanol, 2 mg/L). Volatile compounds (eucalyptol, α-pinene, linalool, eugenol, sabinene) were quantified by normalization to the internal standard.

### 2.7. Statistical Analysis

Each batch of laurel leaf extracts was analyzed in triplicate (*n* = 3), and the entire extraction procedure was performed in duplicate (two separate batches). The Kolmogorov–Smirnov test was employed to evaluate the normality of the data. A one-way analysis of variance (ANOVA) was utilized to determine the statistically significant differences. The findings are presented as means along with their corresponding standard deviations. Partial Least Squares (PLS), Principal Component Analysis (PCA), and Variable Importance Plot (VIP) were applied to assess the correlation among the analyzed parameters.

## 3. Results and Discussion

### 3.1. Selection of Target Analytes

Six volatiles—eucalyptol (1,8-cineole), linalool, α-pinene, β-phellandrene, sabinene, and bornyl acetate—were chosen to represent a chemically diverse and sensorially important segment of the volatile compounds profile. These compounds span key monoterpene classes, including hydrocarbon monoterpenes (α-pinene, sabinene, β-phellandrene), oxygenated monoterpenes (eucalyptol, linalool), and esterified monoterpenoids (bornyl acetate). These compounds were selected according to three crucial factors. First, the analytes exhibit variations in polarity, volatility, and functional groups, facilitating a comprehensive assessment of extraction performance across different compound categories. Moreover, all six compounds exhibit odor-active properties and play a significant role in the overall volatile compounds profile of the sample matrix, thereby serving as valuable indicators for potential improvements in extraction technique. Finally, the compounds produced well-defined peaks with little co-elution, enabling accurate quantification and model fitting.

The chosen analytes featured more distinct chromatographic behavior, enhanced sensory impact, and increased abundance in comparison to other volatile compounds present in the matrix, such as camphene, myrcene, terpinenes, and geraniol. Their inclusion enabled a thorough evaluation of HS-SPME performance and supported the generation of predictive models with a wide range of applications. Furthermore, many of the selected compounds, including eucalyptol, sabinene, and linalool, have been identified as prevalent in laurel leaves [19].

### 3.2. Model Evaluation and Statistical Robustness

Statistical analysis of the RSM models suggested significant reliability and predictive capability for the majority of responses (Table 3). Four out of six models (eucalyptol, α-pinene, sabinene, and β-phellandrene) revealed strong to near-excellent fits, with R^2^ values surpassing 0.93 and adjusted R^2^ values exceeding 0.87. The models for sabinene and β-phellandrene exhibited outstanding performance (R^2^ > 0.999), characterized by low residual error (RMSE < 0.6) and significantly high F-ratios, reflecting superior model accuracy and negligible random variance.

The remaining two models for linalool and bornyl acetate exhibited moderate fits (R^2^ = 0.768 and 0.793, respectively). However, the lack-of-fit tests were non-significant (*p* > 0.05), indicating that the models sufficiently represented the experimental variability. The results indicate that, although the R^2^ values are lower, the models remain statistically appropriate; thus, they can be studied for optimization purposes.

ANOVA reinforced the robustness of the models, as all six responses produced statistically significant F-ratios (*p* < 0.01), indicating that the chosen factors and interactions significantly contribute to the observed variation. When significant two-way interactions (e.g., *X*_2_ × *X*_3_ in eucalyptol) were studied combined with linear and quadratic terms, the identification of curvature and synergistic effects in the response surfaces was revealed.

The HS-SPME models exhibited good predictive performance, with several significant main effects and interactions depending on the analyte. Notably, fiber type and NaCl concentration were among the most influential factors, as confirmed by the ANOVA results (Table S2). The corresponding regression equations (Table S3) describe the multidimensional response surfaces and support the optimization trends discussed in Section 3.3.

Very high R^2^ values (e.g., sabinene, β-phellandrene) resulted from extremely low experimental noise and strong factor effects, as confirmed by duplicated center points and minimal residual variance. For these two analytes, the lack-of-fit test could not be computed because the corresponding models had only one residual degree of freedom, a common situation in sparse-response DOE designs where the compound is not detected across the full experimental domain.

Although the linalool model exhibited a moderate R^2^ value, the lack-of-fit test was non-significant (*p* = 0.084), confirming that the model adequately represents the experimental variability. For linalool and bornyl acetate, the lower R^2^ values reflect the limited variability of these analytes across the experimental domain rather than analytical unreliability.

To strengthen methodological transparency, full ANOVA tables, regression equations, and internal standard stability analysis have been added to the Supplementary Materials. These additions improve reproducibility and support the robustness of the presented models.

### 3.3. Optimization Trends and Practical Implications

The optimal extraction conditions derived from the desirability functions and stepwise regression models are summarized in Table 4. The optimization findings confirmed that most analytes followed the same pattern, which showed that moderate extraction conditions perform well in HS-SPME. The best conditions for four of the six volatiles (eucalyptol, α-pinene, linalool, and bornyl acetate) were a low extraction temperature of 20 °C, no salt (0% NaCl), short equilibration periods of around 10 min, and moderate extraction lengths of 16 to 20 min. The results clearly demonstrate the compound-specific behavior of each fiber: PDMS favored oxygenated monoterpenes such as eucalyptol and bornyl acetate, whereas DVB/CAR/PDMS was superior for monoterpene hydrocarbons including α-pinene and β-phellandrene. These findings reinforce the importance of matching fiber polarity and sorption characteristics to the physicochemical properties of target analytes.

Selection of fiber has been reported to have a major impact on recovery outcomes. Fibers L1 (PDMS) and L3 (DVB/CAR/PDMS) outperformed other fibers. L1 was greatest for eucalyptol, sabinene, and bornyl acetate, while L3 was most effective for α-pinene and β-phellandrene. This indicates that the polarity of the coating and the sorption capacity are critical factors in analyte-fiber interactions, and that choosing a suitable fiber can markedly improve method sensitivity [20].

Sabinene was the sole compound necessitating intermediate conditions (47 °C, 22 min equilibration), suggesting that specific volatiles may gain from enhanced thermal energy and extended exposure durations to achieve equilibrium [21]. This exception underscores the importance of compound-specific optimization and cautions against universal parameter settings.

The narrow confidence intervals around anticipated optima provide further proof that the models are reproducible and robust. The findings offer practical guidance for analysts aiming to optimize the recovery of volatile compounds through HS-SPME and illustrate that RSM can effectively customize extraction protocols based on the physicochemical properties of each target analyte.

**Figure 1:**
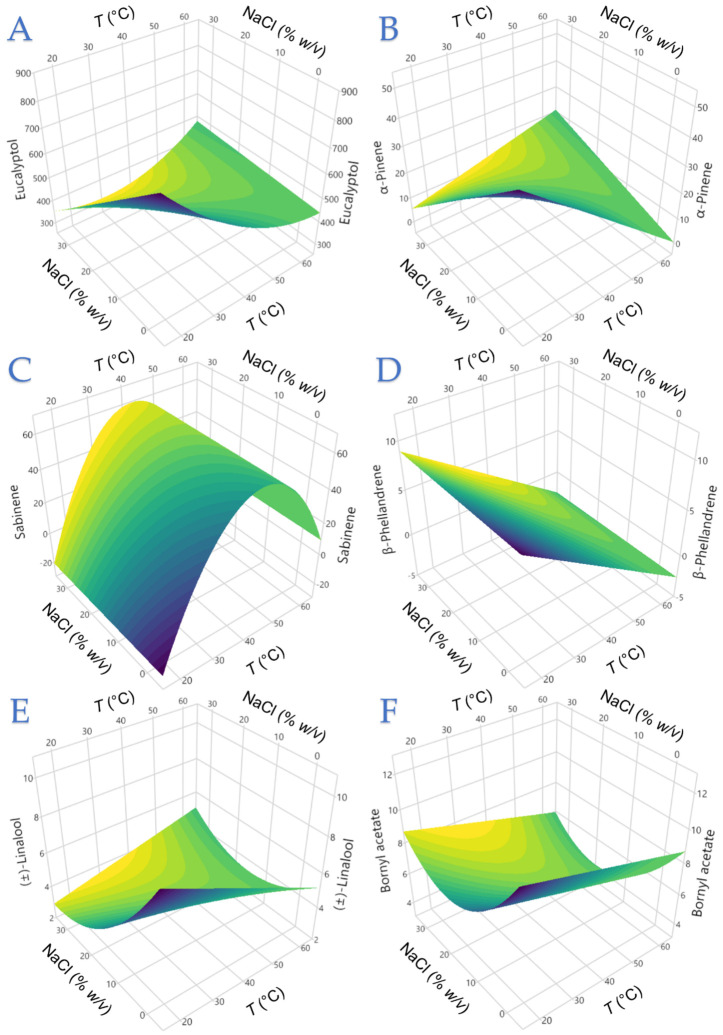
Response surface plots illustrating the interaction between extraction temperature (*X*_2_) and NaCl concentration (*X*_3_) on the predicted semi-quantitative recovery of six key volatiles from *L. nobilis* powder (expressed as mg 2-octanol eq/L). The analytes shown are: (**A**) Eucalyptol, (**B**) α-Pinene, (**C**) Sabinene, (**D**) β-Phellandrene, (**E**) (±)-Linalool, and (**F**) Bornyl acetate.

To visualize the combined effect of extraction temperature and salt concentration, response surface plots for the *X*_2_ × *X*_3_ interaction were generated for each target analyte using the PDMS fiber (L1), which showed the most representative performance across compounds. These surfaces illustrate how thermal and ionic conditions jointly influence volatile recovery under fixed extraction time, sample mass, and stirring rate. As shown in Figure 1, oxygenated monoterpenes (eucalyptol, linalool, bornyl acetate) exhibited clear maxima at moderate temperatures and salt levels, whereas hydrocarbon monoterpenes (α-pinene, sabinene, β-phellandrene) displayed flatter or declining trends at elevated conditions. These patterns confirm that temperature and ionic strength act synergistically for oxygenated compounds, while hydrocarbons are more sensitive to thermal or salting-out stress.

**Figure 2:**
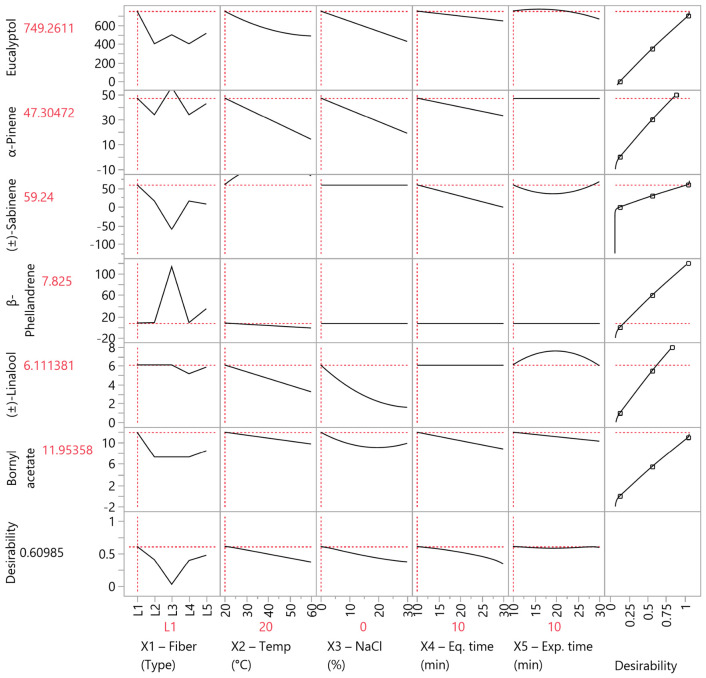
Predictive profiler plots for six volatile compounds and overall desirability. Each subplot shows the predicted response as a function of one experimental factor (*X*_1_–*X*_5_), with all other factors held constant at reference levels (red dotted lines). The red numbers represent the predicted response values at the optimal settings.

Figure 2 presents the predictive profiler, which revealed clear and strong compound-specific trends in the influence of the five experimental factors (*X*_1_–*X*_5_). The most dominant factor was *X*_1_ (fiber type), which produced pronounced differences among coatings. PDMS (L1) yielded the highest predicted recoveries for eucalyptol, sabinene, and bornyl acetate, whereas DVB/CAR/PDMS (L3) was superior for α-pinene, β-phellandrene, and linalool. This pattern confirms that fiber polarity, porosity, and sorption capacity govern analyte–fiber interactions, particularly distinguishing between hydrocarbon and oxygenated monoterpenes. *X*_2_ (temperature) exhibited a curved response, with maximum predicted recoveries at low-to-moderate temperatures (20–30 °C). Beyond this range, recoveries decreased, likely due to reduced partitioning of volatiles into the fiber coating at elevated temperatures. *X*_3_ (NaCl) showed a moderate effect, with slight improvements at low salt levels and plateauing at higher concentrations, indicating a limited salting-out contribution for these monoterpenes. The time-related factors, *X*_4_ (equilibration time) and *X*_5_ (extraction time), had smaller but consistent positive effects, particularly for less volatile compounds such as bornyl acetate and linalool. Increasing extraction time improved predicted recovery up to a point, after which the response leveled off, suggesting that the system approached equilibrium. Finally, the desirability profile demonstrated that the global optimum arises from balancing the individual responses, with the strongest contributions coming from fiber type and temperature. The combined visualization confirms that fiber selection is the primary driver of HS-SPME performance, while temperature fine-tunes the extraction efficiency across compound classes.

### 3.4. Plasma Pretreatment Effects Across Chemically Diverse Volatiles

The RSM analysis of ARTP pretreatment parameters revealed clear response patterns among the six marker volatile compounds, highlighting their intrinsic physicochemical differences. Monoterpene hydrocarbons, such as sabinene, α-pinene, and β-phellandrene, provided better model fits compared to oxygenated monoterpenoids, such as linalool and bornyl acetate. The R^2^ values ranged from 0.92 to 1.00, accompanied by a non-significant lack of fit, suggesting that the response surfaces are both robust and interpretable.

The duration of exposure was recognized as the main factor affecting sabinene and α-pinene, showing significantly negative linear effects, which suggests that prolonged plasma treatment results in a gradual decrease in these highly reactive terpenes. In both instances, duration exhibited a notable interaction with plasma geometry (distance × time) and gas flow (distance × flow), highlighting the influence of local energy density and the residence time of reactive species on the substrate surface. The significant quadratic term in plasma power for sabinene suggests an optimal level at moderate energy input, beyond which over-oxidation or fragmentation takes place.

By contrast, β-phellandrene behaved differently within the hydrocarbon group. Its response was governed primarily by plasma geometry and power, with strong linear effects of distance and power and a pronounced distance × power interaction, while treatment duration did not enter the model. These findings indicate that β-phellandrene responds primarily to spatial energy distribution and substrate thickness rather than to treatment duration, underscoring substantial intra-class variability among closely related monoterpenes.

Oxygenated monoterpenoids showed weaker and noisier responses. For linalool, the model was statistically significant but explained only about half of the observed variance, with a single dominant interaction between distance and gas flow and only marginal quadratic or time-related effects. Bornyl acetate showed similar behavior, with a non-significant overall model yet significant interactions involving distance, flow, and time. These findings suggest that plasma-induced transformations of oxygenated monoterpenoids involve multiple parallel pathways (oxidation, rearrangement, partial hydrolysis) [22], which may not be fully captured by a second-order polynomial model, even though the lack-of-fit remained non-significant.

Taken together, the ARTP-RSM results indicate that plasma pretreatment acts through a combination of treatment time, energy input, and geometric configuration of the discharge, with monoterpene hydrocarbons showing more predictable, time- and power-dependent depletion and oxygenated monoterpenoids displaying more complex, interaction-driven behavior. This mechanistic differentiation is critical for tailoring ARTP conditions towards targeted enhancement or preservation of specific volatile compound attributes in laurel powder and derived food applications.

### 3.5. Model Performance Summary for ARTP–RSM Optimization

The evaluation of the adequacy and predictive performance of the response surface models developed for the five ARTP pretreatment factors was conducted using standard statistical diagnostics. These included the coefficient of determination (R^2^), adjusted R^2^, root mean square error (RMSE), ANOVA F-tests, and lack-of-fit analysis. Table 5 presents the essential model statistics for the six target volatiles.

The models for sabinene, α-pinene, and eucalyptol exhibited satisfactory to excellent performance, with R^2^ values between 0.66 and 0.92, alongside statistically significant ANOVA F-tests (*p* < 0.01). The models accounted for a significant portion of the experimental variance and exhibited a non-significant lack of fit, suggesting that the second-order polynomial structure effectively describes the plasma-induced alterations in these volatiles.

The sabinene model exhibited the highest overall performance (R^2^ = 0.918, Adj-R^2^ = 0.837), indicating significant explanatory power alongside strong lack-of-fit statistics. The α-pinene model demonstrated strong performance (R^2^ = 0.821), exhibiting a balanced distribution of significant linear and interaction terms. The eucalyptol model exhibited a moderate fit (R^2^ = 0.661), with a non-significant lack-of-fit test (*p* = 0.900), indicating that the model structure effectively captured the underlying response trends, notwithstanding increased residual variability.

The model for β-phellandrene provided a near-perfect fit (R^2^ = 0.9997); however, this outcome is influenced by the small sample size (*n* = 8) and the restricted residual degrees of freedom. This model, although statistically valid, should be interpreted with caution and viewed primarily as a high-resolution description of the specific experimental subset rather than as a general predictive model.

Linalool and bornyl acetate proved inferior model performance, with R^2^ values of 0.519 and 0.538, respectively, alongside significantly lower adjusted R^2^ values. The observed responses showed greater intrinsic variability, likely due to the heightened chemical reactivity and multi-pathway transformation characteristics of oxygenated monoterpenoids during plasma exposure. Both models successfully passed the lack-of-fit test (*p* > 0.55), indicating that the observed variability can be attributed to chemical complexity rather than model misspecification.

The statistical diagnostics collectively affirm that the ARTP–RSM framework yielded valid and interpretable models for all six volatiles, displaying particularly strong performance for the monoterpene hydrocarbons. The models successfully identified the key plasma parameters and interactions that influence volatile behavior, thus confirming their utility for optimization and mechanistic interpretation. The models successfully identified the main trends for the more reactive oxygenated compounds; however, they exhibited a higher level of unexplained variance, highlighting the intricacies of their plasma chemistry.

The statistical significance of the key ARTP factors and their interactions is fully documented in Table S5, while the complete regression equations are provided in Table S6. These results collectively confirm that the ARTP–RSM models reliably captured the plasma-induced variability in volatile release, with non-significant lack-of-fit tests supporting the adequacy of the selected second-order structures. Overall, the ARTP optimization framework successfully indicated the dominant operational parameters governing monoterpene and oxygenated monoterpene behavior, thereby reinforcing the mechanistic interpretations presented in this section.

### 3.6. Partial Least Squares (PLS) Regression, Global Optimization, and Experimental Validation

To integrate the multivariate effects of ARTP pretreatment parameters on the overall volatile profile, a Partial Least Squares (PLS) regression model was constructed using all five process variables (*X*_1_–*X*_5_) and the six target volatiles as Y-responses. The NIPALS algorithm with 7-fold cross-validation was applied to capture latent structures linking plasma operating conditions to the combined volatile compounds output.

#### 3.6.1. PLS Model Performance

The PLS model exhibited excellent predictive ability for the multivariate Y-space, explaining 100% of the cumulative Y-variance and 53.4% of the cumulative X-variance across 15 latent factors. Cross-validation showed a progressive improvement in predictive power, with the cumulative Q^2^ increasing steadily to 0.80, indicating strong generalization performance. The minimum Root Mean PRESS (0.386) was obtained at 15 factors, confirming that the volatile responses are governed by a complex, high-dimensional interaction structure consistent with the multifactorial nature of plasma chemistry.

#### 3.6.2. Variable Importance and Drivers of Volatile Compounds Modulation

**Figure 3:**
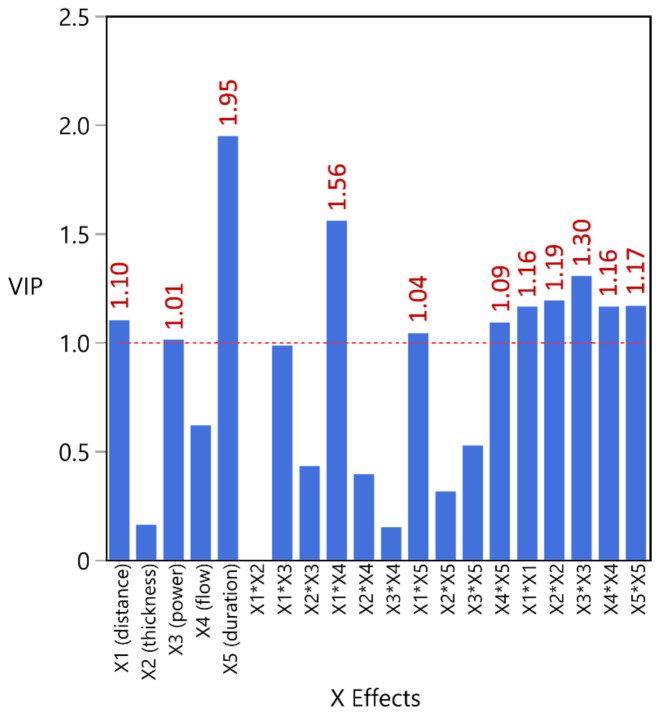
Variable importance in projection (VIP) scores for ARTP parameters and interactions. The red dashed line indicates the VIP threshold (1.0); * is the multiplication symbol for interaction effects.

Variable Importance in Projection (VIP) scores identified the parameters that most significantly contribute to the overall volatile profile. Figure 3 presents the most influential terms (VIP > 1.0). The findings suggest that treatment duration, plasma geometry, and the non-linear (quadratic) effects of power and thickness are the primary global factors influencing the volatile profile. The linear effects alone could not adequately account for the multivariate response, highlighting the fundamentally non-linear characteristics of plasma-matrix interactions.

#### 3.6.3. Identification of Optimal ARTP Conditions

**Figure 4:**
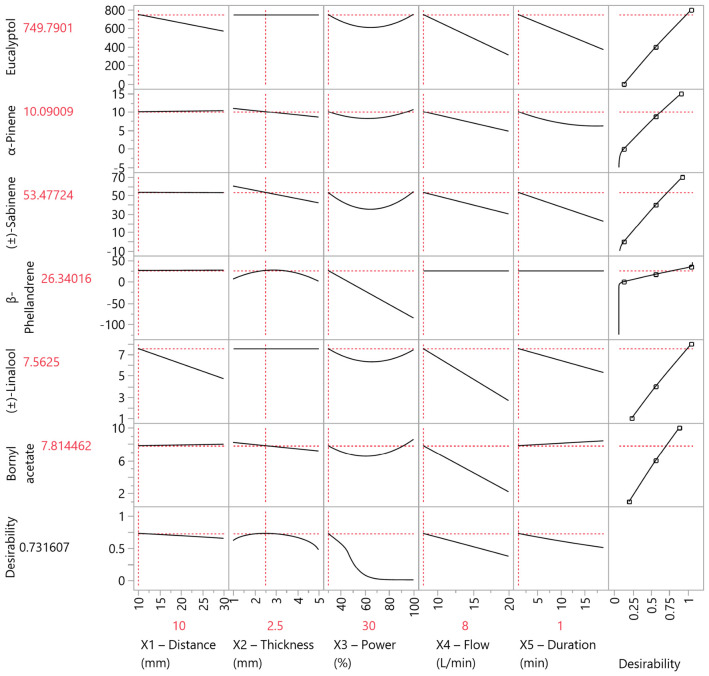
Response profiles of six key volatiles and overall desirability as a function of ARTP parameters (*X*_1_–*X*_5_). PLS regression suggested the optimal values that are noted with red-dashed lines. The red numbers represent the predicted response values at the optimal settings.

A multi-response optimization was conducted utilizing the PLS regression surface to determine the ARTP conditions that concurrently maximize the overall volatile profile. Figure 4 illustrates the global optimum. The specified conditions align with a mild plasma exposure regime, defined by low power, low flow, brief duration, and minimal discharge–sample distance. This configuration reduces over-oxidation and thermal degradation, while improving the release of volatiles from the plant matrix [15]. The anticipated volatile intensities under these optimal conditions were also illustrated in Figure 4. The values indicate the anticipated multi-response optimum obtained from the PLS model.

#### 3.6.4. Model-Based Predictions and Comparison with Untreated Control

The predicted optimum conditions derived from the PLS-RSM models were used to estimate the expected intensities of the six key volatiles, and these model-based predictions were compared with the untreated control to assess the theoretical impact of ARTP pretreatment. Table 6 summarizes the predicted peak areas and the corresponding percent changes relative to the control. The model indicates that mild ARTP pretreatment selectively enhances specific volatile compounds, while reducing others. β-Phellandrene demonstrated a significant increase of 924%, indicating its strong dependence on plasma geometry rather than the duration of treatment [23]. Oxygenated monoterpenoids, such as linalool and bornyl acetate, exhibited significant increases, while monoterpene hydrocarbons, including α-pinene and sabinene, showed decreases, indicating their greater vulnerability to oxidative and radical-driven degradation [24,25].

The values reported in Table 6 represent model-based predicted optima derived from the fitted RSM equations. These conditions were not experimentally validated in the present study, as the primary objective was to establish the optimization framework and identify the dominant plasma parameters rather than to finalize a single operational optimum. Experimental validation of the predicted optima is recommended as a future step for applied process development, particularly for scaling and process integration studies.

#### 3.6.5. Interpretation: ARTP as a Selective Volatile-Compound-Modulating Pretreatment

The integration of PLS optimization and experimental validation demonstrates that ARTP does not uniformly enhance all volatiles; rather, it selectively modifies the volatile compounds’ profile. Under mild plasma conditions, the treatment results in: (I) an increase in eucalyptol, β-phellandrene, linalool, and bornyl acetate; (II) a decrease in α-pinene and sabinene. This pattern indicates a deliberate transition from a profile rich in green, resinous monoterpene hydrocarbons to one characterized by eucalyptol, exhibiting a shift toward oxygenated monoterpenoids, which in the literature are associated with different odor descriptors, although no sensory evaluation was performed in this study. This selective modulation underscores ARTP as a tunable pretreatment strategy, capable of customizing the sensory attributes of laurel powder and potentially improving its applicability for specific food uses.

#### 3.6.6. Comprehensive Volatile Profiling Under Optimal ARTP Conditions

A comprehensive GC–MS profiling was conducted to evaluate the overall effect of ARTP pretreatment on the entire volatile fraction of laurel powder, in addition to the six marker volatiles analyzed through RSM and PLS (Table 7). The expanded dataset demonstrated extensive and selective modulation across various chemical classes, indicating that ARTP affects not only the targeted compounds but also the broader volatile compound matrix. Similar patterns were observed in other studies [26,27]. Compound identification was confirmed through mass spectral matching and retention indices (RI, Kovats Index), calculated using a C7–C30 *n*-alkane standard (Supelco 49451-U) under identical chromatographic conditions.

Under optimal ARTP conditions (10 mm distance, 2.5 mm thickness, 30% power, 8 L/min flow, 1 min duration), monoterpene hydrocarbons revealed a heterogeneous response. It is clear from Table 7 that some volatile compounds present on laurel leaves were completely gone after the ARTP process, while there were also some compounds detected on the final ARTP sample but not in the initial one. Several highly reactive hydrocarbons exhibited significant decreases, including α-pinene (–65.0%), sabinene (–21.0%), β-pinene, γ-terpinene, and 3-carene, which align with their vulnerability to oxidation, radical-driven degradation, and rearrangement during plasma exposure. In contrast, specific hydrocarbons such as β-phellandrene (+924.1%) and tricyclene (new) exhibited significant increases, indicating enhanced release from the plant matrix or selective stabilization under mild plasma conditions.

A clearer trend was noted for oxygenated monoterpenoids, which generally exhibited an increase following ARTP treatment. Compounds including eucalyptol (+18.2%), terpinen-4-ol (+49.7%), α-terpineol (+47.7%), linalool (+87.2%), and pinocarveol (novel) suggested significant enhancement. The observed increases suggest that mild plasma exposure facilitates the easier release of oxygenated volatiles, and also their easier release from bound precursors or their synthesis via regulated oxidative pathways. This can be attributed to the lack of reactive oxygen species in the plasma matrix, as the operating gas implemented was nitrogen. Thus, the RNS may cause the oxidation of unsaturated chemical bonds in the volatile compounds, leading to the formation of more stable, saturated ones [28,29]. This pattern corresponds with the PLS findings, indicating that oxygenated monoterpenoids were significantly positively influenced by the global optimum.

Ester compounds exhibited significant increases, with bornyl acetate rising by 51.3%, α-terpinyl acetate by 61.6%, neryl acetate by 273.0%, and linalyl anthranilate by 42.4%. The esters are reported in the literature to contribute to various odor notes, but their sensory relevance in this study was not evaluated. Their enhancement indicates that ARTP may promote ester release or diminish competing degradation pathways [30]. The extent of these increases underscores the significant possibility of ARTP to enhance high-impact volatile compounds.

Several sesquiterpenes, including α-selinene, γ-elemene, aromandendrene, and humulene, along with the phenylpropanoid methyleugenol, were exclusively observed following ARTP treatment. Their emergence suggests that plasma exposure may activate deeper structural components of the plant matrix, facilitating the detection of compounds that are either absent or below detection limits in untreated samples. This supports the hypothesis that ARTP induces microstructural changes that improve volatile accessibility and may initiate mild rearrangement reactions. The ARTP-optimal sample demonstrates nearly double the concentration of eucalyptol compared to studies that did not subject laurel leaves to plasma treatment [6].

To assess the applicability of these effects to actual food systems, the same analysis was conducted using a broth model. The trends identified in the powder were predominantly maintained and, in certain instances, intensified. Oxygenated monoterpenoids and esters exhibited significant increases in broth, specifically eucalyptol (+92.5%), terpinen-4-ol (+108.8%), α-terpineol (+212.1%), bornyl acetate (+140.3%), and α-terpinyl acetate (+120.4%). Some compounds exhibited significant increases, such as terpinolene (+1343.3%), while others were exclusively present in the broth following ARTP, including δ-cadinene. The findings indicate that ARTP-induced modifications remain effective during aqueous dispersion and thermal processing, thereby affirming their applicability in food contexts. The literature reports that ARTP is emerging as a new technique for increasing the yield of bioactive compounds from foods [31]. Moreover, similar results have been observed in previous studies, where ARTP has been successfully applied to increase the extraction yield of bioactive compounds from rosemary [32] and *Moringa oleifera* [15] leaves. It should be noted that the optimized HS-SPME conditions were developed using laurel powder, whereas the broth matrix contains significantly higher water content, which can alter headspace partitioning. Therefore, broth results should be interpreted as qualitative indicators of ARTP-induced changes rather than a direct quantitative extension of the powder optimization.

The comprehensive profiling indicates that ARTP functions as a selective volatile-compound-modulating pretreatment, altering the volatile composition from reactive monoterpene hydrocarbons to oxygenated monoterpenoids, esters, and sesquiterpenes, which possess greater sensory impact. The changes extend beyond analytical extracts to food-relevant environments, highlighting the potential of ARTP to modulate the volatile composition of laurel powder, with potential implications for aroma that require future sensory validation for culinary and industrial uses.

#### 3.6.7. Principal Component Analysis (PCA) of the Complete Volatile Dataset

**Figure 5:**
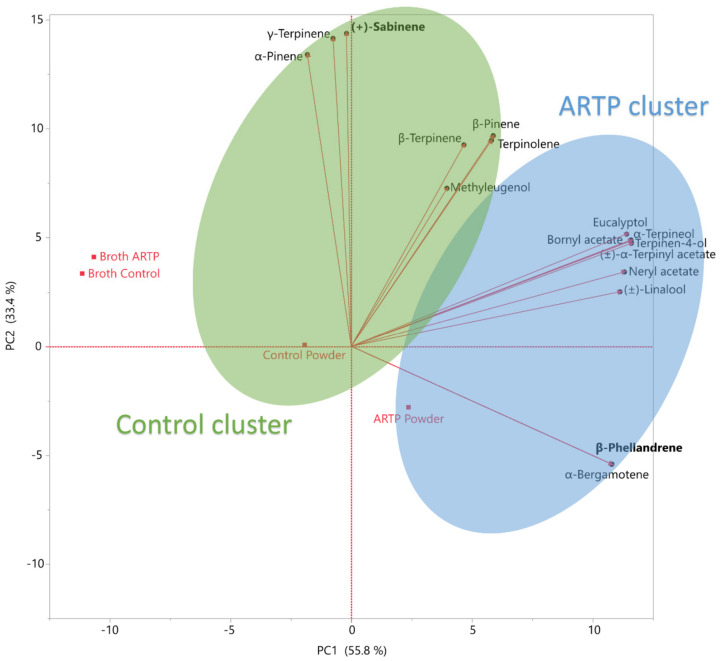
PCA biplot of volatile profiles across control and ARTP-treated laurel powder and broth samples. Principal Component 1 (PC1, 55.8%) separates samples based on oxygenated monoterpenoids and esters, while PC2 (33.4%) reflects hydrocarbon and matrix effects. Control samples (green) cluster together and are associated with sabinene, α-pinene, and γ-terpinene. ARTP-treated samples (blue) shift toward eucalyptol, bornyl acetate, α-terpineol, and linalool, indicating selective volatile compounds modulation. Vectors represent compound loadings and highlight the chemical drivers of sample separation. The red dashed line indicates the zero-loading reference for PC1 and PC2.

To visualize the multivariate impact of ARTP pretreatment across both powder and broth matrices, Principal Component Analysis (PCA) was performed using the full volatile dataset (Figure 5). The first two principal components explained 89.2% of the total variance (PC1 = 55.8%, PC2 = 33.4%), indicating a highly structured model with strong discriminatory power.

PC1 was positively associated with oxygenated monoterpenoids, esters, and several sesquiterpenes, including eucalyptol, terpinen-4-ol, α-terpineol, (±)-linalool, bornyl acetate, α-terpinyl acetate, neryl acetate, linalyl anthranilate, 4-thujen-2α-yl acetate, caryophyllene, humulene, aromadendrene, α-bergamotene, and α-guaiene. In addition, β-pinene, β-terpinene, and terpinolene also loaded positively, indicating that not all hydrocarbons decreased under ARTP treatment. Negative loadings on PC1 were restricted to α-pinene, (±)-sabinene, and γ-terpinene, which characterize the untreated control samples. Overall, PC1 reflects a transition from a hydrocarbon-rich, green-resinous profile toward a composition enriched in oxygenated monoterpenoids and esters, which have been linked in prior studies to different odor descriptors.

PC2 further classified samples according to the relative abundance of reactive hydrocarbons and matrix-sensitive sesquiterpenes. Positive loadings were identified for α-pinene, (±)-sabinene, γ-terpinene, and methyleugenol, suggesting that these compounds exhibit significant variability in relation to matrix effects and thermal processing. Negative loadings correlated with β-phellandrene, pinocarvone, linalyl anthranilate, 4-thujen-2α-yl acetate, bornyl acetate, and various sesquiterpenes, including caryophyllene, humulene, aromadendrene, and α-bergamotene. This component records secondary volatile compounds changes and the retention or loss of volatiles dependent on the matrix, especially under aqueous and thermal conditions [33].

The PCA score plot identified four distinct sample clusters: (i) Control Powder and Broth Control clustered in the negative PC1 region, indicating a common baseline characterized by monoterpene hydrocarbons. (ii) ARTP Powder samples exhibited a significant shift toward the positive PC1 direction, indicating a considerable enrichment in oxygenated volatiles and esters. (iii) Broth ARTP samples, despite being shifted leftward due to dilution and heating, maintained elevated levels along PC2 compared to Broth Control, indicating selective retention and enhancement of essential volatiles within a food-relevant matrix.

The observed patterns indicate that ARTP pretreatment leads to a significant, directional, and matrix-independent alteration in the volatile profile, aligning with the trends identified in RSM, PLS, and comprehensive profiling. The PCA effectively demonstrates the selective volatile-compound-modulating ability of ARTP, distinguishing between control and treated samples in chemical aspects only, as no sensory analysis was conducted.

The integrated analysis of RSM, PLS, and PCA offers a clear mechanistic insight into the modulation of the volatile profile of laurel by ARTP pretreatment. RSM identified optimal plasma conditions that maximized key oxygenated monoterpenoids. PLS regression confirmed that these compounds—specifically eucalyptol, terpinen-4-ol, α-terpineol, and bornyl acetate—were the most significant positive contributors to the global optimum. PCA confirmed this directional shift, demonstrating a distinct separation of ARTP-treated samples along PC1 towards a cluster enriched in oxygenated monoterpenoids, esters, and specific sesquiterpenes, whereas control samples were associated with monoterpene hydrocarbons including α-pinene, sabinene, and γ-terpinene. The multivariate approaches collectively indicate that ARTP does not produce random or diffuse chemical alterations; rather, it facilitates a selective, predictable, and matrix-independent transformation of the volatile profile towards compounds with enhanced sensory impact. The convergence of statistical frameworks reinforces the conclusion that ARTP can be effectively utilized as a volatile-compound-modulating pretreatment in both culinary and industrial contexts.

The optimization of ARTP parameters provided a statistically robust framework for predicting how plasma-induced changes influence the release of key laurel volatiles. However, while the response surfaces identify which combinations of distance, power, flow, thickness, and duration enhance or suppress specific compounds, they do not explain why these effects occur. Understanding the underlying mechanisms—such as the selective enhancement of oxygenated monoterpenes at moderate intensities or the decline of hydrocarbon monoterpenes under prolonged exposure—requires consideration of the physicochemical interactions between plasma species and the plant microstructure. To address this, the following sections provide a mechanistic interpretation of ARTP–matrix interactions and integrate these insights with the compound-specific responses observed in this study.

### 3.7. Mechanistic Interpretation of ARTP Effects

The compound-specific responses observed in the ARTP optimization can be interpreted through the interactions between plasma-generated species and the plant microstructure [34]. Atmospheric plasma produces a mixture of reactive oxygen species (ROS), reactive nitrogen species (RNS), charged particles, and UV photons, which interact with epidermal tissues and induce surface micro-etching, cell-wall erosion, and localized weakening of cuticular and pectin layers [35]. These structural modifications increase the accessibility of intracellular oil glands and secretory structures, thereby enhancing the release of volatile constituents during HS-SPME. Although these effects are well documented in other aromatic plants, their influence on laurel powder has not been previously clarified.

Several studies on aromatic herbs, such as green tea leaves, peppermint, and hyssop, support these mechanisms [26]. In the current study, moderate ARTP intensities increased the recovery of eucalyptol, linalool, and bornyl acetate, all of which are oxygenated monoterpenes typically stored deeper within secretory structures. Their enhanced release suggests that ARTP facilitates diffusion by opening micro-channels in the leaf-powder matrix and partially disrupting cuticular waxes and pectin-rich domains. This pattern aligns with earlier observations in plasma-treated basil leaves, where ROS-mediated membrane oxidation increased permeability [36]. The consistency across different plant matrices strengthens the interpretation that moderate plasma exposure primarily promotes structural loosening rather than destructive effects.

In contrast, α-pinene, sabinene, and β-phellandrene, which are hydrocarbon monoterpenes, showed strong sensitivity to plasma geometry and exposure duration, with prolonged treatment leading to reduced recovery. These compounds are more volatile and more susceptible to plasma-induced desorption or mild oxidative loss. Their decline at higher intensities is not surprising, as similar behavior has been reported in thyme and rosemary, where cold plasma caused micro-fracturing of glandular trichomes and partial volatilization of monoterpenes [37,38]. The significant distance × duration and distance × flow interactions found in our models further support the roles of local energy density and residence time of reactive species in determining whether plasma exposure enhances or suppresses volatile release. In practical terms, this means that even small deviations in plasma geometry can shift the balance between beneficial micro-etching and unwanted volatilization.

The behavior of β-phellandrene is particularly illustrative: its maximum recovery occurred at a short distance and a short duration, suggesting that brief, high-energy micro-etching is sufficient to rupture superficial oil glands without inducing volatilization losses. This mirrors findings in plasma-treated leaves, where increased porosity and surface erosion were observed at low-to-moderate intensities [39]. Such compound-specific optima highlight the importance of considering both volatility and anatomical localization when interpreting plasma effects.

Overall, the mechanistic evidence supports a model in which ARTP enhances volatile release primarily through surface erosion, micro-scale structural opening, and ROS/RNS-driven weakening of cell-wall components. The compound-specific responses observed here reflect differences in molecular polarity, volatility, and localization within the leaf microstructure. This helps explain why oxygenated monoterpenes benefit from moderate ARTP intensities, while hydrocarbon monoterpenes require more carefully controlled exposure to avoid losses. This mechanistic understanding strengthens the interpretation of the RSM models and provides a scientific basis for tailoring ARTP conditions to specific classes of volatile compounds.

**Figure 6:**
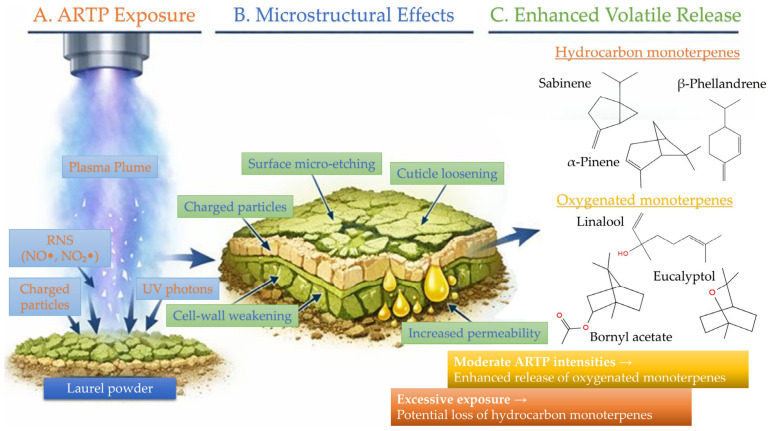
Conceptual mechanism of ARTP-induced microstructural modifications in laurel powder. Base illustration generated with ChatGPT(GPT‑5.5 Instant); final layout, annotations, and graphical composition were manually edited in Microsoft PowerPoint.

These mechanistic insights are visually summarized in Figure 6, which illustrates the sequence of ARTP exposure, microstructural modifications, and compound-specific volatile release. The schematic highlights how reactive nitrogen species, charged particles, and UV photons interact with the powdered laurel matrix, inducing surface micro-etching, cuticle loosening, and cell-wall weakening. These changes facilitate the release of oxygenated monoterpenes at moderate intensities, while excessive exposure may lead to partial loss of hydrocarbon monoterpenes due to volatilization or oxidative degradation. This visual summary complements the experimental findings and reinforces the class-dependent nature of ARTP effects.

It should be noted that the optimized HS-SPME conditions were developed using laurel powder, whereas the broth matrix contains significantly higher water content, which can alter headspace partitioning. Therefore, broth results should be interpreted as qualitative indicators of ARTP-induced changes rather than a direct quantitative extension of the powder optimization. Interpretation of plasma-induced mechanisms has been limited to trends directly supported by the data. No claims regarding aroma modulation or sensory impact are made, as sensory evaluation was not performed.

### 3.8. Integrated Discussion

The combined evaluation of HS-SPME optimization, ARTP pretreatment, and food-model validation provides a comprehensive understanding of how analytical conditions and plasma-induced microstructural changes jointly determine the release of laurel volatiles. The HS-SPME models established that fiber type and extraction temperature were the dominant analytical factors. These findings highlight the importance of matching fiber polarity and sorption characteristics to the physicochemical properties of target analytes, and they provide the analytical foundation for interpreting the effects of ARTP pretreatment. It is worth noting that these analytical trends were consistent across most compounds, reinforcing the robustness of the extraction framework.

The ARTP optimization revealed that plasma exposure can significantly modulate volatile availability by altering the microstructure of the powdered leaf matrix. The enhancement of eucalyptol and bornyl acetate, both oxygenated monoterpenes, at moderate intensities suggests that ARTP facilitates volatile release primarily through surface micro-etching, loosening of pectin-rich domains, and increased permeability of the plant tissue. Sabinene, although a hydrocarbon monoterpene, also showed improved recovery under similar conditions, indicating that certain hydrocarbons may benefit from controlled micro-etching before volatilization losses become significant. These effects are consistent with previous reports of cold-plasma-induced micro-fracturing of glandular trichomes in oregano and thyme [37], cell-wall thinning in rosemary [38], and ROS-mediated membrane oxidation in basil [36]. In practical terms, this means that ARTP does not act uniformly across compounds but instead interacts with their volatility and anatomical localization.

**Figure 7:**
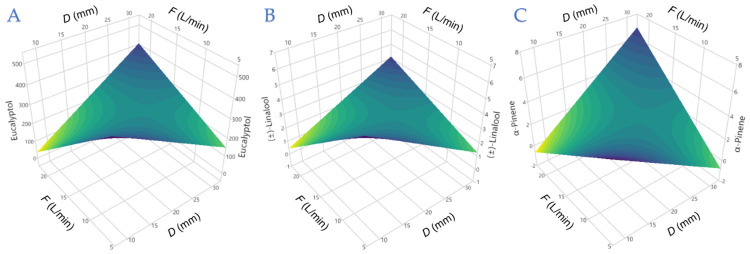
Response surface plots illustrating the interaction between distance (*X*_1_) and nitrogen flow rate (*X*_4_) on the predicted semi-quantitative recovery (mg 2-octanol eq/L) of selected volatiles from ARTP-treated *L. nobilis* powder. Panels illustrate the contrasting behavior of oxygenated monoterpenes ((**A**): Eucalyptol, (**B**): Linalool) and a hydrocarbon monoterpene ((**C**): α-Pinene) under varying plasma geometry and reactive species delivery.

To illustrate the compound-class-specific effects of plasma geometry and reactive species delivery, response surface plots for the distance × nitrogen flow rate interaction (*X*_1_ × *X*_4_) were generated for three representative volatiles. As shown in Figure 7, oxygenated monoterpenes such as eucalyptol and linalool exhibited clear optima at intermediate distances and moderate flow rates, whereas the hydrocarbon monoterpene α-pinene showed reduced recovery under close-range or high-flow conditions.

Hydrocarbon monoterpenes such as α-pinene and β-phellandrene exhibited a more complex response, with improvements at short exposure times and reduced recovery under prolonged or high-power conditions. Oxygenated monoterpenes benefit from moderate intensities that open diffusion pathways, whereas hydrocarbons require carefully controlled exposure to avoid volatilization losses.

The broth model provided a practical validation of these mechanistic and analytical insights. ARTP-treated laurel powder produced higher headspace concentrations of key volatile compounds under realistic culinary conditions, demonstrating that plasma-induced microstructural changes translate into sensory-relevant improvements. This observation is particularly important from an application standpoint, as enhanced volatile compound transfer can improve flavor intensity without increasing dosage or relying on synthetic additives. The non-thermal nature of ARTP and the use of nitrogen as a process gas further support its suitability for heat-sensitive phytochemicals and clean-label formulations.

Taken together, the integrated results show that ARTP pretreatment and optimized HS-SPME conditions act synergistically: HS-SPME maximizes analytical recovery, while ARTP increases the intrinsic availability of volatiles within the plant matrix. This dual-optimization approach provides a robust framework for enhancing the extraction and sensory performance of aromatic plant materials. The mechanistic understanding gained here also offers a foundation for scaling ARTP to industrial applications and for extending the approach to other herbs, spices, and botanical ingredients.

This study provides the first comprehensive evaluation of ARTP pretreatment on the volatile profile of *L. nobilis* powder and is the first to integrate plasma-induced microstructural modification with optimized HS-SPME/GC-MS analysis. Unlike previous plasma studies on aromatic herbs, which focused primarily on microbial inactivation or phenolic extraction, the present work demonstrates that ARTP can selectively modulate the release of both hydrocarbon and oxygenated monoterpenes in a powdered botanical matrix. The inclusion of a ready-to-use broth model further strengthens the practical relevance of the findings, showing that ARTP-enhanced volatile compounds release is not limited to analytical conditions but extends to real food systems. Together, these advances position ARTP as a novel, non-thermal, and clean-label pretreatment strategy for improving the sensory performance of culinary herbs and natural flavoring agents.

However, this research is primarily focused on the quantitative chemical and physical characterization of the material. Consequently, a key limitation is the lack of corresponding sensory data. While parameters such as volatile compounds or texture profile analysis can be predictive of sensory attributes, they are not a substitute for direct human evaluation. The subjective experience of attributes like taste, odor, and texture, which ultimately dictate consumer preference, was not assessed. Future work should integrate this analysis to correlate the instrumental findings with human perception.

To further validate the postulated mechanisms of action, future investigations should use high-resolution microscopy to visually document the physical alterations induced in laurel powder by ARTP treatment. Scanning electron microscopy (SEM) would be essential in furnishing tangible evidence of surface micro-etching, enabling a comparison viewing of the originally smooth or undamaged plant cell walls versus the roughened, pitted, and disturbed surfaces after treatment. Simultaneously, transmission electron microscopy (TEM) may provide a more profound understanding of the interior structural effects, revealing nuanced alterations in porosity and the degree of delamination or fracture inside the cell wall layers. Atomic force microscopy (AFM) might enhance these results by enabling a quantitative assessment of nanoscale topography changes and surface roughness. Such imaging data would surpass indirect biochemical experiments, providing strong visual evidence that the improved extractability and bioactivity of laurel volatile compounds are directly associated with the diminished physical integrity of the biomass.

The optimal conditions predicted by the RSM models were not experimentally validated in the present study. This was a deliberate methodological choice, as the primary objective was to establish the optimization framework and identify the most influential factors rather than to finalize a single operational optimum. Future work will include confirmatory experiments at the predicted optima to validate the model outputs and support potential scale-up of the ARTP pretreatment.

This study acknowledges a limitation in the direct visualization of the physical effects of ARTP on laurel powder. While the observed increases in extraction yields and bioactivity strongly imply enhanced permeability and surface erosion, we were unable to perform direct microscopic analyses, such as SEM, TEM, or AFM, to confirm these structural modifications. The absence of such imaging data means that proposed mechanisms, including surface micro-etching, increased porosity, and partial disruption of cell wall layers, remain inferential rather than definitively proven. Future investigations should prioritize these advanced microscopy techniques to provide visual confirmation of these structural changes and to establish a more concrete link between ARTP-induced physical alterations and the observed improvements in volatile extractability.

## 4. Conclusions

This study provides the first integrated evaluation of Atmospheric Room Temperature Plasma (ARTP) as a pretreatment for enhancing the volatile profile of *L. nobilis* powder. By combining HS-SPME optimization with plasma-induced microstructural modification, the work demonstrates that analytical conditions and plasma parameters jointly shape the recovery of key volatile compounds. A central finding is that oxygenated monoterpenes respond positively to moderate plasma intensities, whereas hydrocarbon monoterpenes require more controlled exposure to avoid volatilization losses. These compound-specific behaviors reflect differences in volatility, polarity, and anatomical localization within the leaf matrix. The ready-to-use broth model confirmed that ARTP-treated laurel powder delivers higher headspace concentrations of characteristic volatiles under realistic culinary conditions. This practical outcome suggests that ARTP can enhance volatile compound transfer without increasing dosage, offering a clean-label approach for improving flavor intensity in food applications. The non-thermal nature of the process and the use of nitrogen as a process gas further support its suitability for heat-sensitive phytochemicals. Overall, the findings highlight ARTP as a promising pretreatment strategy for aromatic herbs and natural flavoring agents. The mechanistic insights gained here may guide future efforts to scale ARTP for industrial use, and the dual-optimization framework presented in this study can be extended to other botanical materials. The findings describe relative changes in headspace volatile responses and do not imply direct sensory outcomes.

## Figures and Tables

**Table 1:** Experimental factors and levels used in the HS-SPME/GC-MS design.

Factor	Type	Levels (Coded)	Actual Values
Fiber type (*X*_1_)	Categorical (5 levels)	L1–L5	PDMS, PDMS/DVB, DVB/CAR/PDMS, CAR/PDMS, PA
Extraction temperature (*X*_2_)	Continuous	−1, 0, +1	20, 40, 60 °C
NaCl concentration (*X*_3_)	Continuous	−1, 0, +1	0, 15, 30% (*w*/*v*)
Equilibration time (*X*_4_)	Continuous	−1, 0, +1	10, 20, 30 min
Extraction time (*X*_5_)	Continuous	−1, 0, +1	10, 20, 30 min

**Table 2:** The independent variables that were used to enhance the ARTP pretreatment were given by their actual values and coded levels.

Independent Variables	Coded Units	Coded Levels		
		−1	0	1
Distance (*D*, mm)	*X*_1_	10	20	30
Thickness (*T*, mm)	*X*_2_	1	3	5
Power (*P*, %)	*X*_3_	30	65	100
Flow (*F*, L/min)	*X*_4_	8	14	20
Duration (*t*, min)	*X*_5_	1	10	19

**Table 3:** Statistical performance metrics of RSM models for the six marker volatiles.

Statistic	Eucalyptol	α-Pinene	(±)-Sabinene	β-Phellandrene	(±)-Linalool	Bornyl Acetate
R^2^	0.933	0.932	0.999	0.999	0.768	0.793
Adj-R^2^	0.873	0.899	0.999	0.999	0.620	0.690
RMSE	68.36	5.45	0.585	0.205	1.17	1.67
Mean Response	178.68	11.58	12.77	27.49	3.21	3.19
Observations (*n*)	20	19	10	5	19	19
Model DF/Error DF	9/10	6/12	8/1	3/1	7/11	6/12
F-ratio (ANOVA)	15.49	27.61	937.6	73,004.50	5.2	7.67
*p*-value (ANOVA)	<0.0001	<0.0001	0.0253	0.0027	0.0079	0.0015
Lack-of-Fit (F, *p*)	F = 1.66, *p* = 0.367 (ns)	F = 1.99, *p* = 0.310 (ns)	–	–	F = 6.02, *p* = 0.084 (ns)	F = 1.47, *p* = 0.472 (ns)
Max R^2^	0.986	0.99	–	–	0.986	0.975

“ns” denotes non-significant.

**Table 4:** Optimal HS-SPME conditions per analyte based on desirability and stepwise regression predictions.

Compound	Optimal Fiber (*X*_1_)	Temp (*X*_2_, °C)	NaCl (*X*_3_, *w*/*v*)	Eq Time (*X*_4_, min)	Ext Time (*X*_5_, min)	Desirability	Predicted Recovery (Stepwise Regression)
Eucalyptol	PDMS (L1)	20	0	10	16	0.997	769.72 ± 137.95
α-Pinene	DVB/CAR/PDMS (L3)	20	0	10	—	0.917	55.98 ± 9.10
(±)-Sabinene	PDMS (L1)	47	—	22	19	0.938	57.29 ± 7.10
β-Phellandrene	DVB/CAR/PDMS (L3)	20	—	—	—	0.927	113.21 ± 2.61
(±)-Linalool	DVB/CAR/PDMS (L3)	20	0	—	20	0.721	7.60 ± 1.84
Bornyl acetate	PDMS (L1)	20	0.4	10	20	0.981	10.99 ± 2.68

Values represent the predicted semi-quantitative recovery (mg 2-octanol eq/L). “—” indicates that the factor was not influential in the optimal solution.

**Table 5:** Statistical performance metrics of ARTP–RSM models for the six marker volatiles.

Statistic	Eucalyptol	α-Pinene	(±)-Sabinene	β-Phellandrene	(±)-Linalool	Bornyl Acetate
R^2^	0.661	0.821	0.918	0.9997	0.519	0.538
Adj-R^2^	0.518	0.668	0.837	0.9989	0.317	0.22
RMSE	142.08	2.346	8.505	0.349	1.657	2.531
Mean Response	360.37	5.23	22.48	6.9	3.77	5.59
Observations (*n*)	28	27	27	8	28	28
Model DF/Error DF	8/19	12/14	13/13	5/2	8/19	11/16
F-ratio (ANOVA)	4.63	5.37	11.27	1238.95	2.57	1.69
*p*-value (ANOVA)	0.0029	0.0020	<0.0001	0.0008	0.0438	0.1642 (ns)
Lack-of-Fit (F, *p*)	0.406, *p* = 0.900 (ns)	8.89, *p* = 0.257 (ns)	1.60, *p* = 0.556 (ns)	–	0.321, *p* = 0.944 (ns)	1.59, *p* = 0.560 (ns)
Max R^2^	0.893	0.999	0.996	–	0.823	0.981

“ns” denotes non-significant.

**Table 6:** Model-predicted intensities of six key volatiles under optimal ARTP conditions compared to untreated control.

Analyte	PLS Regression	ARTP Optimal	Control	% Change
Eucalyptol	749.79	760.01 ± 17.48 *	643.2 ± 18.65	18.2%
α-Pinene	10.09	9 ± 0.52	25.7 ± 1.16 *	–65.0%
(±)-Sabinene	53.48	57.99 ± 1.22	73.44 ± 3.75 *	–21.0%
β-Phellandrene	26.34	6.25 ± 0.43 *	0.61 ± 0.02	924.1%
(±)-Linalool	7.56	8.09 ± 0.31 *	4.32 ± 0.17	87.2%
Bornyl acetate	7.81	11.16 ± 0.46 *	7.37 ± 0.16	51.3%

Values indicate model-predicted peak areas derived from the fitted RSM equations. Percent change reflects the predicted relative increase or decrease compared to the untreated control. The values are expressed as the mean ± standard deviation (mg/L). “PLS Regression” refers to the predicted response at the global optimum obtained from the PLS model. “ARTP Optimal” and “Control” refer to the experimentally measured concentrations in laurel powder, both with and without ARTP pretreatment, respectively. Asterisks (*) indicate statistically significant differences between ARTP and control samples (*p* < 0.05).

**Table 7:** Comprehensive volatile compound profile of laurel powder and broth samples with and without ARTP pretreatment.

Compound	RI	Chemical Group	Control Powder	ARTP Powder	% Change	Broth Control	Broth ARTP	% Change
α-Pinene	923	Monoterpene hydrocarbon	25.7 ± 1.16 *	9 ± 0.52	−65.0	0.2 ± 0.01	0.3 ± 0.01 *	50.8
(+)-Sabinene	958	Bicyclic monoterpene hydrocarbon	73.44 ± 3.75 *	57.99 ± 1.22	−21.0	0.85 ± 0.02	0.95 ± 0.06	11.2
β-Pinene	959	Bicyclic monoterpene hydrocarbon	7.19 ± 0.17	n.d.		0.26 ± 0.01	0.29 ± 0.02	11.0
β-Terpinene	981	Cyclic monoterpene hydrocarbon	n.d.	n.d.		0.33 ± 0.01	0.37 ± 0.01 *	12.5
β-Phellandrene	982	Monoterpene hydrocarbon	0.61 ± 0.02	6.25 ± 0.43 *	924.1	n.d.	n.d.	
Tricyclene	990	Monoterpene hydrocarbon	n.d.	1.84 ± 0.14	new	n.d.	n.d.	
Eucalyptol	1017	Monoterpenoid ether	643.2 ± 18.65	760.01 ± 17.48 *	18.2	17.63 ± 1.16	33.94 ± 0.92 *	92.5
2-Carene	1071	Bicyclic monoterpene hydrocarbon	n.d.	n.d.		0.51 ± 0.02	n.d.	
γ-Terpinene	1075	Monoterpene hydrocarbon	7.53 ± 0.47 *	5.12 ± 0.15	−31.9	0.98 ± 0.06	1.83 ± 0.08 *	87.4
Terpinolene	1083	Monoterpene hydrocarbon	3.62 ± 0.09	n.d.		0.09 ± 0	1.24 ± 0.09 *	1343.3
(±)-Linalool	1083	Monoterpenoid alcohol	4.32 ± 0.17	8.09 ± 0.31 *	87.2	n.d.	0.39 ± 0.02	new
*trans*-(-)-Pinocarveol	1110	Bicyclic monoterpenoid alcohol	n.d.	0.83 ± 0.02	new	n.d.	n.d.	
Pinocarvone	1124	Bicyclic monoterpenoid ketone	1.46 ± 0.11	1.59 ± 0.11	9.0	n.d.	n.d.	
Terpinen-4-ol	1151	Monoterpenoid alcohol	8.36 ± 0.23	12.51 ± 0.38 *	49.7	0.57 ± 0.04	1.19 ± 0.09 *	108.8
α-Terpineol	1163	Monoterpenoid alcohol	3.32 ± 0.11	4.91 ± 0.14 *	47.7	0.21 ± 0.01	0.66 ± 0.05 *	212.1
3-Carene	1200	Bicyclic monoterpene hydrocarbon	2.25 ± 0.07	n.d.		n.d.	n.d.	
4-Carene	1232	Bicyclic monoterpene hydrocarbon	0.48 ± 0.02	n.d.		n.d.	n.d.	
Linalyl anthranilate	1240	Μonoterpenoid ester	0.59 ± 0.03	0.84 ± 0.04 *	42.4	n.d.	n.d.	
4-Thujen-2α-yl acetate	1252	Bicyclic monoterpenoid ester	0.78 ± 0.05	1.23 ± 0.05 *	58.5	n.d.	n.d.	
Bornyl acetate	1259	Bicyclic monoterpenoid ester	7.37 ± 0.16	11.16 ± 0.46 *	51.3	0.63 ± 0.02	1.52 ± 0.07 *	140.3
Bornylene	1276	Bicyclic monoterpene hydrocarbon	0.68 ± 0.04	n.d.		n.d.	n.d.	
(±)-α-Terpinyl acetate	1328	Monoterpenoid ester	131.81 ± 5.27	213.05 ± 11.5 *	61.6	12.11 ± 0.53	26.69 ± 1.2 *	120.4
Neryl acetate	1343	Monoterpenoid ester	1.58 ± 0.05	5.89 ± 0.24 *	273.0	0.15 ± 0.01	0.37 ± 0.03 *	154.1
Ylangene	1354	Tricyclic sesquiterpene hydrocarbon	1.98 ± 0.14	n.d.		n.d.	n.d.	
Methyleugenol	1370	Phenylpropanoid	n.d.	22.73 ± 0.82	new	0.43 ± 0.03	n.d.	
β-Elemene	1373	Sesquiterpene hydrocarbon	14.3 ± 0.57	n.d.		n.d.	n.d.	
Caryophyllene	1393	Bicyclic sesquiterpene hydrocarbon	3.06 ± 0.08	5.04 ± 0.31 *	64.6	n.d.	n.d.	
α-Guaiene	1418	Sesquiterpene hydrocarbon	1.14 ± 0.04	1.67 ± 0.1 *	46.7	n.d.	n.d.	
Humulene	1425	Monocyclic sesquiterpene hydrocarbon	1.17 ± 0.03	2.08 ± 0.09 *	77.9	n.d.	n.d.	
Aromandendrene	1432	Tricyclic sesquiterpene hydrocarbon	0.61 ± 0.03	1.02 ± 0.03 *	67.9	n.d.	n.d.	
Germacrene	1452	Sesquiterpene hydrocarbon	3.95 ± 0.1	n.d.		n.d.	n.d.	
α-Selinene	1456	Sesquiterpene hydrocarbon	n.d.	6.22 ± 0.27	new	n.d.	n.d.	
γ-Elemene	1468	Sesquiterpene hydrocarbon	n.d.	2.69 ± 0.01	new	n.d.	n.d.	
Viridiflorene	1468	Tricyclic sesquiterpene hydrocarbon	1.72 ± 0.01	n.d.		n.d.	n.d.	
α-Bergamotene	1480	Sesquiterpene hydrocarbon	3.11 ± 0.19	5.12 ± 0.2 *	64.7	n.d.	n.d.	
δ-Cadinene	1497	Bicyclic sesquiterpene hydrocarbon	n.d.	n.d.		n.d.	0.32 ± 0.01	new

Mean concentrations (mg/L ± SD) of all detected volatile compounds in laurel powder solutions (Control vs. Optimal ARTP) and in broth prepared from the same material (Broth Control vs. Broth ARTP). Percent change indicates the relative increase or decrease induced by ARTP pretreatment. “n.d.” denotes compounds not detected, while “new” indicates compounds detected exclusively after ARTP exposure. Asterisks (*) denote statistically significant differences between treatments (*p* < 0.05). RI denote Retention indices (Kovats Index) based on C7–C30 *n*-alkanes.

## Data Availability

The original contributions presented in this study are included in the article/Supplementary Materials. Further inquiries can be directed to the corresponding author.

## Supplementary Materials

The following supporting information can be downloaded at https://www.mdpi.com/article/10.3390/foods15132346/s1. Table S1. HS-SPME DOE design matrix with merged coded/actual factor levels and semi-quantitative responses. Table S2. ANOVA *p*-values for HS-SPME RSM models. Table S3. Regression equations (coded units) for HS-SPME RSM models. Table S4: ARTP CCD matrix with coded and actual factor levels and semi-quantitative responses. Table S5. ANOVA *p*-values for ARTP RSM models. Table S6. Regression equations (coded units) for ARTP RSM models.
