# Supplementary material for: Effect of Atmospheric Room Temperature Plasma on the Volatile Profile of Laurel: Optimization by HS-SPME/GC-MS Analysis with Application in a Ready-to-Use Broth Model
Source: Foods. 2026 Jul 2;15(13):2346. doi: 10.3390/foods15132346 (PMC13362320; doi:10.3390/foods15132346)
Supplement: Supplementary file 1 [file foods-15-02346-s001.zip › foods-4393315-supplementary.pdf]

Supplementary Materials

Effect of Atmospheric Room Temperature Plasma on the Volatile Profile of Laurel: Optimization by HS-SPME/GC-MS Analysis with Application in a Ready-to-Use Broth Model

Martha Mantiniotou [1], Vassilis Athanasiadis [1], Dimitrios Kalompatsios [1], Eleni Bozinou [1], George Ntourtoglou [2], Vassilis G. Dourtoglou [2] and Stavros I. Lalas [1,*]

[1] Department of Food Science and Nutrition, University of Thessaly, Terma N. Temponera Street, 43100 Karditsa, Greece; mmantiniotou@uth.gr (M.M.); vaathanasiadis@uth.gr (V.A.); dkalompatsios@uth.gr (D.K.); empozinou@uth.gr (E.B.)
[2] Department of Wine, Vine and Beverage Sciences, University of West Attica, Agiou Spyridonos Street, Egaleo, 12210 Athens, Greece; gntourtoglou@uniwa.gr (G.N.); vdourt@uniwa.gr (V.G.D.)
* Correspondence: slalas@uth.gr; Tel.: +30−24410−64783

Table S1. HS-SPME DOE design matrix with merged coded/actual factor levels and semi-quantitative responses.

Run	Coded/actual factor levels					Actual responses (mg 2-octanol eq/L)					
	X_1 - Fiber (Type)	X_2 - Temp (°C)	X_3 - NaCl (%)	X_4 - Eq. Time (min)	X_5 - Ext. Time (min)	Eucalyptol	α-Pinene	(±)-Sabinene	β-Phellandrene	(±)-Linalool	Bornyl acetate
1	L3 (DVB/CAR/PDMS)	0 (40)	1 (30)	−1 (10)	1 (30)	42.87	n.d.	1.24	n.d.	1.98	0.94
2	L4 (CAR/PDMS)	0 (40)	0 (15)	0 (20)	0 (20)	50.66	2.07	n.d.	3.93	2.24	0.99
3	L5 (PA)	−1 (20)	1 (30)	−1 (10)	−1 (10)	20.28	0.83	2.92	n.d.	1.54	7.8
4	L2 (PDMS/DVB)	−1 (20)	0 (15)	1 (30)	1 (30)	83.03	3.55	n.d.	8.43	1.93	1.28
5	L5 (PA)	0 (40)	0 (15)	0 (20)	0 (20)	35.55	0.61	2.29	n.d.	2.35	0.87
6	L2 (PDMS/DVB)	1 (60)	1 (30)	0 (20)	−1 (10)	86.98	3.68	10.11	n.d.	2.93	1.59
7	L4 (CAR/PDMS)	1 (60)	0 (15)	−1 (10)	1 (30)	23.26	0.57	n.d.	n.d.	1.09	0.61
8	L4 (CAR/PDMS)	0 (40)	−1 (0)	1 (30)	−1 (10)	119.03	3.43	8.83	n.d.	3.92	1.82
9	L1 (PDMS)	0 (40)	0 (15)	0 (20)	0 (20)	412.86	16.78	n.d.	3.18	4.24	4.48
10	L2 (PDMS/DVB)	1 (60)	−1 (0)	−1 (10)	0 (20)	149.46	4.03	15.65	n.d.	4.12	4.7
11	L3 (DVB/CAR/PDMS)	−1 (20)	0 (15)	−1 (10)	−1 (10)	439.95	45.64	n.d.	n.d.	n.d.	2.96
12	L4 (CAR/PDMS)	−1 (20)	1 (30)	0 (20)	0 (20)	50.96	4.89	n.d.	8.72	1.09	0.5
13	L5 (PA)	1 (60)	−1 (0)	0 (20)	1 (30)	75.09	0.92	5.07	n.d.	3.85	1.3
14	L3 (DVB/CAR/PDMS)	−1 (20)	−1 (0)	0 (20)	0 (20)	437.69	51.93	n.d.	113.21	9.1	6.28
15	L1 (PDMS)	−1 (20)	1 (30)	1 (30)	1 (30)	215.72	3.09	7.44	n.d.	1.96	4.01
16	L5 (PA)	1 (60)	1 (30)	1 (30)	0 (20)	38	1.27	n.d.	n.d.	2.69	1.04
17	L2 (PDMS/DVB)	0 (40)	0 (15)	0 (20)	0 (20)	97.26	4.87	15.54	n.d.	4.08	n.d.

18	L1 (PDMS)	0 (40)	0 (15)	0 (20)	0 (20)	535.65	25.92	58.61	n.d.	5.3	7.31
19	L3 (DVB/CAR/PDMS)	1 (60)	−1 (0)	1 (30)	−1 (10)	52.61	1.58	n.d.	n.d.	1.9	1.12
20	L1 (PDMS)	−1 (20)	−1 (0)	−1 (10)	1 (30)	606.74	44.35	n.d.	n.d.	4.69	10.92

''n.d.'' denotes not detected.

Table S2. ANOVA *p*-values for HS-SPME RSM models.

Compound	Term	*p*-value	Significant ($p < 0.05$)
Eucalyptol	X_1{L5&L4&L2 – L3&L1}	<0.0001	Yes
	X_1{L3 – L1}	0.0008	Yes
	X_2	0.0266	Yes
	X_3	0.0023	Yes
	X_4	0.032	Yes
	X_5	0.0842	No
	$X_2 \times X_3$	0.0055	Yes
	X_2^2	0.1358	No
	X_5^2	0.141	No
α-Pinene	X_1{L5&L4&L2 – L1&L3}	<0.0001	Yes
	X_1{L1 – L3}	0.068	No
	X_2	0.0016	Yes
	X_3	0.0318	Yes
	X_4	0.0015	Yes
	$X_2 \times X_3$	0.0001	Yes
(±)-Sabinene	X_1{L3&L5&L4&L2 – L1}	0.01	Yes
	X_1{L3&L5 – L4&L2}	0.0258	Yes
	X_1{L3 – L5}	0.0243	Yes
	X_2	0.019	Yes
	X_4	0.0196	Yes
	X_5	0.0901	No
	X_2^2	0.0189	Yes
	X_5^2	0.0297	Yes
β-Phellandrene	X_1{L1&L4&L2 – L3}	0.0018	Yes
	X_1{L1 – L4&L2}	0.2349	No
	X_2	0.0344	Yes
(±)-Linalool	X_1	0.127	No
	X_2	0.2266	No
	X3	0.0036	Yes
	X_5	0.9212	No
	X_3^2	0.1356	No
	X_5^2	0.027	Yes
	$X_2 \times X_3$	0.0275	Yes
Bornyl acetate	X_1	0.0007	Yes
	X_2	0.0448	Yes

	X_3	0.0551	No (trend)
	X_4	0.0095	Yes
	X_5	0.1394	No
	X_3^2	0.06	No (trend)

Table S3. Regression equations (coded units) for HS-SPME RSM models.

$Y_{Eucalyptol} = 198.344 - 111.112 \cdot X_1\{L5\&L4\&L2 - L3\&L1\} - 126.269 \cdot X_1\{L3 - L1\} - 54.053 \cdot X_2 - 83.163 \cdot X_3 - 52.450 \cdot X_4 - 42.689 \cdot X_5 + 57.515 \cdot X_2^2 - 54.843 \cdot X_5^2 + 78.510 \cdot (X_2 \cdot X_3)$	(S1)
$Y_{\alpha\text{-Pinene}} = 14.566 - 8.862 \cdot X_1\{L5\&L4\&L2 - L1\&L3\} - 4.337 \cdot X_1\{L1 - L3\} - 6.888 \cdot X_2 - 4.483 \cdot X_3 - 7.113 \cdot X_4 + 9.730 \cdot (X_2 \cdot X_3)$	(S2)
$Y_{Sabinene} = 25.796 - 32.814 \cdot X_1\{L3\&L5\&L4\&L2 - L1\} - 22.265 \cdot X_1\{L3\&L5 - L4\&L2\} - 31.573 \cdot X_1\{L3 - L5\} + 11.973 \cdot X_2 - 30.005 \cdot X_4 + 4.105 \cdot X_5 - 41.283 \cdot X_2^2 + 27.985 \cdot X_5^2$	(S3)
$Y_{\beta\text{-Phellandrene}} = 56.060 - 52.505 \cdot X_1\{L1\&L4\&L2 - L3\} - 0.375 \cdot X_1\{L1 - L4\&L2\} - 4.645 \cdot X_2$	(S4)
$Y_{Linalool} = 3.414 - 0.470 \cdot X_1 - 0.453 \cdot X_2 - 1.280 \cdot X_3 - 0.041 \cdot X_5 + 1.004 \cdot X_3^2 - 1.528 \cdot X_5^2 + 0.979 \cdot (X_2 \cdot X_3)$	(S5)
$Y_{Bornyl\ acetate} = 3.380 - 2.332 \cdot X_1 - 1.105 \cdot X_2 - 1.039 \cdot X_3 - 1.577 \cdot X_4 - 0.844 \cdot X_5 + 1.677 \cdot X_3^2$	(S6)

Table S4. ARTP CCD design matrix with coded and actual factor levels and semi-quantitative responses.

Run	Coded/actual factor levels					Actual responses (mg 2-octanol eq/L)					
	X_1 - Distance (mm)	X_2 - Thickness (mm)	X_3 - Power (%)	X_4 - Flow (L/min)	X_5 - Duration (min)	Eucalyptol	α-Pinene	(±)-Sabinene	β-Phellandrene	(±)-Linalool	Bornyl acetate
1	−1 (10)	1 (5)	1 (100)	1 (20)	1 (19)	287.97	2.69	10.7	n.d.	3.44	5.79
2	−1 (10)	−1 (1)	−1 (30)	−1 (8)	−1 (1)	780.49	11.95	64.77	6.04	7.76	8.9
3	0 (20)	1 (5)	0 (65)	0 (14)	0 (10)	57.07	0.76	3.17	n.d.	0.66	0.89
4	−1 (10)	−1 (1)	−1 (30)	1 (20)	1 (19)	357.37	5.05	18.97	n.d.	4.17	6.76
5	−1 (10)	0 (3)	0 (65)	0 (14)	0 (10)	67.01	0.61	2.16	n.d.	0.93	1.07
6	1 (30)	1 (5)	−1 (30)	1 (20)	1 (19)	346.62	3.25	18.88	1.73	3.51	6.48
7	0 (20)	0 (3)	1 (100)	0 (14)	0 (10)	400.26	4.26	17.46	n.d.	4.6	7.62
8	0 (20)	−1 (1)	0 (65)	0 (14)	0 (10)	512.93	6.22	23.4	n.d.	6.48	9.39
9	1 (30)	1 (5)	1 (100)	−1 (8)	1 (19)	77.82	n.d.	2.97	n.d.	0.85	1.37
10	1 (30)	0 (3)	0 (65)	0 (14)	0 (10)	431.38	6.17	n.d.	30.67	4.7	7.17
11	0 (20)	0 (3)	−1 (30)	0 (14)	0 (10)	337.82	5.21	29.62	n.d.	3.15	4.71
12	0 (20)	0 (3)	0 (65)	0 (14)	0 (10)	344.05	2.59	16.23	n.d.	3.59	5.73
13	1 (30)	−1 (1)	1 (100)	−1 (8)	−1 (1)	637.08	11.26	54.89	n.d.	5.34	9.21
14	0 (20)	0 (3)	0 (65)	0 (14)	1 (19)	360.72	3.07	1.92	n.d.	3.83	6.85
15	−1 (10)	−1 (1)	1 (100)	−1 (8)	1 (19)	195.81	2.65	9.85	n.d.	2.99	3.81
16	−1 (10)	1 (5)	−1 (30)	−1 (8)	1 (19)	518.92	4.71	13.41	n.d.	7.65	11.39
17	1 (30)	−1 (1)	−1 (30)	−1 (8)	1 (19)	71.12	0.73	2.47	n.d.	1.12	1.28
18	−1 (10)	1 (5)	−1 (30)	1 (20)	−1 (1)	341.92	7.38	34.01	0.98	3.07	4.03
19	0 (20)	0 (3)	0 (65)	0 (14)	0 (10)	163.13	1.44	6.58	0.58	1.83	2.86
20	1 (30)	1 (5)	1 (100)	1 (20)	−1 (1)	646.8	16.2	70.03	n.d.	5.34	8.95
21	1 (30)	−1 (1)	1 (100)	1 (20)	1 (19)	285.18	2.36	0.79	12.31	3.79	5.56
22	0 (20)	0 (3)	0 (65)	−1 (8)	0 (10)	266.31	2.63	11.66	n.d.	3.74	5.24

23	0 (20)	0 (3)	0 (65)	0 (14)	−1 (1)	314.54	4.36	14.92	1.27	2.47	3.23
24	0 (20)	0 (3)	0 (65)	1 (20)	0 (10)	171.45	1.69	9.17	n.d.	1.78	2.84
25	−1 (10)	−1 (1)	1 (100)	1 (20)	−1 (1)	203.23	3.12	14.58	n.d.	1.6	2.58
26	−1 (10)	1 (5)	1 (100)	−1 (8)	−1 (1)	750.92	11	58.15	n.d.	7.1	8.18
27	1 (30)	−1 (1)	−1 (30)	1 (20)	−1 (1)	596.59	11.86	53.89	n.d.	5.34	7.56
28	1 (30)	1 (5)	−1 (30)	−1 (8)	−1 (1)	565.73	7.95	42.37	1.62	4.71	7.1

''n.d.'' denotes not detected.

Table S5. ANOVA *p*-values for ARTP RSM models.

Compound	Term	*p*-value	Significant ($p < 0.05$)
Eucalyptol	X_1	0.8002	No
	X_3	0.4828	No
	X_4	0.3113	No
	X_5	0.001	Yes
	$X_1 \times X_4$	0.012	Yes
	$X_3 \times X_5$	0.4895	No
	$X_4 \times X_5$	0.0273	Yes
	X_3^2	0.02	Yes
α-Pinene	X_1	0.5679	No
	X_2	0.6086	No
	X_3	0.42	No
	X_4	0.6432	No
	X_5	<0.0001	Yes
	$X_1 \times X_4$	0.0103	Yes
	$X_1 \times X_5$	0.0193	Yes
	$X_2 \times X_4$	0.179	No
	$X_3 \times X_5$	0.2284	No
	$X_4 \times X_5$	0.2156	No
	X_3^2	0.1091	No
	X_5^2	0.375	No
(±)-Sabinene	X_1	0.4123	No
	X_2	0.7844	No
	X_3	0.2998	No
	X_4	0.428	No
	X_5	<0.0001	Yes
	$X_1 \times X_4$	0.007	Yes
	$X_2 \times X_3$	0.0169	Yes
	$X_1 \times X_5$	0.0403	Yes
	$X_4 \times X_5$	0.0659	No (trend)
	$X_2 \times X_4$	0.0989	No
	$X_3 \times X_4$	0.357	No
	$X_3 \times X_5$	0.3636	No
	X_3^2	0.0001	Yes

β-Phellandrene	X_1	0.0002	Yes
	X_2	0.0094	Yes
	X_3	0.0003	Yes
	$X_1 \times X_3$	0.0003	Yes
	X_2^2	0.0003	Yes
(±)-Linalool	X_1	0.575	No
	X_3	0.4493	No
	X_4	0.2052	No
	X_5	0.1219	No
	$X_1 \times X_4$	0.0093	Yes
	$X_3 \times X_5$	0.5651	No
	$X_4 \times X_5$	0.0895	No (trend)
	X_3^2	0.0831	No (trend)
Bornyl acetate	X_1	0.8424	No
	X_2	0.9364	No
	X_3	0.6386	No
	X_4	0.5884	No
	X_5	0.3449	No
	$X_1 \times X_4$	0.0394	Yes
	$X_1 \times X_5$	0.0435	Yes
	$X_2 \times X_5$	0.4631	No
	$X_3 \times X_5$	0.3058	No
	$X_4 \times X_5$	0.1123	No
	X_3^2	0.1168	No

Table S6. Regression equations (coded units) for ARTP RSM models.

$$Y_{\mathrm{Eucalyptol}} = 268.859 + 8.593 \cdot X_1 - 23.973 \cdot X_3 - 34.837 \cdot X_4 - 129.765 \cdot X_5 + 98.693 \cdot (X_1 \cdot X_4) - 25.034 \cdot (X_3 \cdot X_5) + 84.947 \cdot (X_4 \cdot X_5) + 142.344 \cdot X_3^2 \quad \text{(S7)}$$

$$Y_{\alpha\text{-Pinene}} = 2.719 + 0.348 \cdot X_1 - 0.312 \cdot X_2 - 0.495 \cdot X_3 + 0.282 \cdot X_4 - 3.607 \cdot X_5 + 1.885 \cdot (X_1 \cdot X_4) + 0.901 \cdot (X_2 \cdot X_4) - 1.683 \cdot (X_1 \cdot X_5) - 0.802 \cdot (X_3 \cdot X_5) + 0.826 \cdot (X_4 \cdot X_5) + 2.196 \cdot X_3^2 + 1.176 \cdot X_5^2 \quad \text{(S8)}$$

$$Y_{\mathrm{Sabinene}} = 10.107 + 1.753 \cdot X_1 + 0.560 \cdot X_2 - 2.165 \cdot X_3 - 1.640 \cdot X_4 - 18.203 \cdot X_5 + 5.823 \cdot (X_2 \cdot X_3) + 6.801 \cdot (X_1 \cdot X_4) + 3.779 \cdot (X_2 \cdot X_4) - 2.031 \cdot (X_3 \cdot X_4) - 4.843 \cdot (X_1 \cdot X_5) - 2.002 \cdot (X_3 \cdot X_5) + 4.269 \cdot (X_4 \cdot X_5) + 18.660 \cdot X_3^2 \quad \text{(S9)}$$

$$Y_{\beta\text{-Phellandrene}} = 0.925 + 29.745 \cdot X_1 - 2.530 \cdot X_2 - 26.610 \cdot X_3 + 29.398 \cdot (X_1 \cdot X_3) - 23.678 \cdot X_2^2 \quad \text{(S10)}$$

$$Y_{\mathrm{Linalool}} = 3.001 - 0.223 \cdot X_1 - 0.302 \cdot X_3 - 0.512 \cdot X_4 - 0.632 \cdot X_5 + 1.199 \cdot (X_1 \cdot X_4) - 0.243 \cdot (X_3 \cdot X_5) + 0.741 \cdot (X_4 \cdot X_5) + 1.195 \cdot X_3^2 \quad \text{(S11)}$$

$$Y_{\mathrm{Bornyl\ acetate}} = 4.527 + 0.121 \cdot X_1 - 0.048 \cdot X_2 - 0.286 \cdot X_3 - 0.329 \cdot X_4 - 0.581 \cdot X_5 + 1.419 \cdot (X_1 \cdot X_4) - 1.387 \cdot (X_1 \cdot X_5) + 0.476 \cdot (X_2 \cdot X_5) - 0.669 \cdot (X_3 \cdot X_5) + 1.063 \cdot (X_4 \cdot X_5) + 1.655 \cdot X_3^2 \quad \text{(S12)}$$
